# Modulation of Host Cell Death and Lysis Are Required for the Release of *Simkania negevensis*

**DOI:** 10.3389/fcimb.2020.594932

**Published:** 2020-10-29

**Authors:** Rebecca-Diana Koch, Eva-Maria Hörner, Nadine Münch, Elke Maier, Vera Kozjak-Pavlovic

**Affiliations:** Department of Microbiology, Biocenter, University of Würzburg, Würzburg, Germany

**Keywords:** *Simkania negevensis*, exit, release, cell death, caspases

## Abstract

*Simkania negevensis* is a *Chlamydia*-like bacterium and emerging pathogen of the respiratory tract. It is an obligate intracellular bacterium with a biphasic developmental cycle, which replicates in a wide range of host cells. The life cycle of *S. negevensis* has been shown to proceed for more than 12 days, but little is known about the mechanisms that mediate the cellular release of these bacteria. This study focuses on the investigation of host cell exit by *S. negevensis* and its connection to host cell death modulation. We show that *Simkania*-infected epithelial HeLa as well as macrophage-like THP-1 cells reduce in number during the course of infection. At the same time, the infectivity of the cell culture supernatant increases, starting at the day 3 for HeLa and day 4 for THP-1 cells and reaching maximum at day 5 post infection. This correlates with the ability of *S. negevensis* to block TNFα-, but not staurosporin-induced cell death up to 3 days post infection, after which cell death is boosted by the presence of bacteria. Mitochondrial permeabilization through Bax and Bak is not essential for host cell lysis and release of *S. negevensis*. The inhibition of caspases by Z-VAD-FMK, caspase 1 by Ac-YVAD-CMK, and proteases significantly reduces the number of released infectious particles. In addition, the inhibition of myosin II by blebbistatin also strongly affects *Simkania* release, pointing to a possible double mechanism of exit through host cell lysis and potentially extrusion.

## Introduction

*Simkania negevensis* is a *Chlamydia*-like microorganism, first described as a contaminant in cell cultures in 1993 (Kahane et al., 1993). It is a gram-negative, obligate intracellular bacterium, with a wide host range from amoeba to mammalian cells (Kahane et al., 2001). *S. negevensis* has been considered to be an emerging pathogen due to the connection with respiratory tract infections, such as community-acquired pneumonia (Lieberman et al., 1997), bronchiolitis in infants (Kahane et al., 1998), and acute rejection in lung recipients (Husain et al., 2007). This association between *S. negevensis* and respiratory diseases has been recently challenged (Al-Younes et al., 2017). In addition, an increased seropositivity and the presence of *S. negevensis* DNA in biopsies of Crohn’s disease patients have been shown (Scaioli et al., 2019).

The life cycle of *S. negevensis* resembles the one of *Chlamydia*, with an infective form represented by smaller, electron-dense equivalents of chlamydial elementary bodies (EBs), and the replicative, metabolically active form being present as reticulated, homogenously stained bodies (RBs), which re-differentiate into EBs (Kahane et al., 2002). *S. negevensis* develop in a *Simkania*-containing vacuole (SnCV), a membranous system that forms intimate contacts with the endoplasmic reticulum (ER) and mitochondria during infection of human cells (Mehlitz et al., 2014). The development proceeds significantly longer than is the case with *Chlamydia*. It has been reported that the number of RBs in the SnCV reaches the maximum three days after infection, when the re-differentiation into EBs starts, which can continue over the next 12 days without increase in infectivity or notable cell lysis (Kahane et al., 2002).

Cell death plays a critical role in the life cycle of pathogenic bacteria and viruses, where its inhibition or induction can benefit bacterial survival, immune system evasion or release from infected cells and transmission to the new host (Rudel et al., 2010). Major cell death mechanisms include programmed cell death or apoptosis, inflammatory cell death or pyroptosis, and necrosis, all of which can be influenced by pathogens (Fink and Cookson, 2005; Jorgensen et al., 2017). Apoptosis and pyroptosis both rely on the activation of cysteinyl-aspartate specific proteases (caspases). Apoptosis can be induced by intrinsic and extrinsic signals. As a response to intrinsic stresses, the intrinsic pathway of apoptosis is activated, which results in permeabilization of mitochondrial outer membrane through oligomerization of Bax and Bak proteins, release of cytochrome *c* and activation of initiator caspase 9. Extrinsic pathway of apoptosis is induced through stimulation of death receptors, such as tumor necrosis factor receptor (TNFR) by death ligands, which include TNFα. This results in the activation of initiator caspase 8. In both cases downstream effector caspases 3, 6, and 7 are subsequently activated. Pyroptosis, on the other hand, is triggered by so-called inflammasomes, which are cytosolic sensors that respond to a variety of signals and activate caspase 1. This results in the cleavage of various interleukins and gasdermin D, which ends in the permeabilization of the plasma membrane and cell lysis (Jorgensen et al., 2017). *Chlamydia* effectively manipulate apoptosis during their development (Byrne and Ojcius, 2004). *S. negevensis* is also capable of suppressing the ER stress response and inhibiting apoptosis, at least during the initial phases of infection (Karunakaran et al., 2011; Mehlitz et al., 2014).

One of the most important steps in the life cycle of intracellular bacteria is the release or exit from the infected host cell (Flieger et al., 2018). Whereas exit strategies of certain pathogenic microorganisms, including related *Chlamydia trachomatis* have been the focus of several studies, very little is known about the release of *S. negevensis*. *C. trachomatis* has been reported to leave infected cells by a dual pathway. On one side, the host cell is lysed with the help of proteases to enable the release of chlamydial EBs, a process that reaches its maximum 72 h post infection. On the other side, chlamydial infective particles exit through a process called extrusion, with the involvement of actin polymerization, neuronal Wiskott–Aldrich syndrome protein (N-WASP), myosin II and Rho GTPase. Interestingly, these release mechanisms appear to be conserved among different *Chlamydia* species (Hybiske and Stephens, 2007). For *S. negevensis* it has been shown that there is a significant increase in the number of infective particles in the infected cell culture supernatant between day 2 and day 3 post infection, with further increase at the later stages of infection (day 6, 9 and 14). This was accompanied by an increase in cell mortality but only after day 9 post infection, an effect that strongly depended on the cell type used (Vouga et al., 2017).

Considering different observations about the development of *S. negevensis* reported by various studies and the general lack of knowledge concerning the release of *S. negevensis* from infected cells, we were interested in studying these processes in more detail. Contrary to several previous publications, we could observe progressive loss of *S. negevensis*-infected epithelial cells starting from day 4 post infection, which coincided with a massive release of infectious progeny on days 4 and 5. We could connect these observations to the inhibition of cell death by *S. negevensis*, which ceased to be effective at the same time when the increased cell loss in infected culture was observed. Finally, we show the involvement of caspases, and in particular caspase 1, as well as proteases and myosin II in the release of *S. negevensis* from infected cells, similar to what has been described for the related microorganism *C. trachomatis*. Our data, therefore, offer interesting first insights into the so far unexplored process of the *S. negevensis* exit from infected host cells.

## Material and Methods

### Cell Culture and Bacteria

HeLa (ATCC^®^ CCL-2.1™) and THP-1 (ATCC^®^ TIB-202™) cells were grown in RPMI1640 medium (Thermo Fisher Scientific, Dreieich, Germany) supplemented with 10% FCS (Sigma/Merck, Darmstadt, Germany). For differentiation of THP-1 cells into macrophages, 5 × 10^5^ cells were seeded into a 12-well plate and treated with 20 ng/ml phorbol 12-myristate 13-acetate (PMA) (Sigma/Merck, Darmstadt, Germany) for 72 h. HeLa cells with a knockout of Bax and Bak or overexpressing Bcl-XL were a kind gift from A. Weber and were generated as described before (Weber et al., 2016; Brokatzky et al., 2019). For *S. negevensis* preparation HeLa cells were grown to 50-60% confluence and infected in infection medium (RPMI w/o HEPES supplemented with 5% heat inactivated FCS) at MOI 1 for 6 h at 35°C, 5% CO_2_. Medium was then replaced by fresh infection medium and infected cells were grown for 3 days. Cells were mechanically detached, and bacteria released using ~ 2–5 mm glass beads (Carl Roth, Karlsruhe, Germany). Low speed supernatant (600 × g) was subjected to high-speed centrifugation (20,000 × g) to pellet bacteria. Bacteria were washed with 5 ml SPG buffer [250 mM sucrose, 4 mM monopotassium phosphate, 10 mM disodium phosphate, and 5 mM glutamate (pH 7.4)], aliquoted and stored at -80°C in the SPG buffer. Work with *S. negevensis* was conducted in a biosafety level 2 laboratory registered with the Government of Lower Franconia under code 55.1-8791.1.30.

### Infection, Inhibitors, and Re-Infection Experiments

HeLa cells were grown to 50–60% confluence and THP-1 cells were differentiated into macrophage-like cells using PMA. The cells were infected in infection medium with *S. negevensis* at MOI 1 for 6 h at 35°C, 5% CO_2_, after which the medium was replaced by fresh infection medium. For inhibitor studies, in case of Hela cells, the inhibitors were added to the medium at this point. In case of THP-cells, inhibitors were added 3 days later. For re-infection experiments, 24 h old medium of infected cells was transferred to fresh HeLa cells, which were then incubated at 35°C and 5% CO_2_ for 24 h when the medium was changed with the fresh infection medium. 48 h later, the cells were fixed for immunofluorescence or lysed for western blot analysis. For western blot analysis of the supernatants, the infectious particles were isolated by centrifugation at 14,000 × g and lysed in Laemmli buffer [62.5 mM Tris (pH 6.8), 2% SDS, 10% glycerol, 5% ß-mercaptoethanol, and 0.002% Bromophenol Blue].

### Apoptosis Induction

Infected and non-infected HeLa cells were treated with 25 ng/ml TNFα (BD Biosciences, Heidelberg, Germany) and 5 µg/ml of cycloheximide (CHX) (Sigma/Merck, Darmstadt, Germany), for 12 h or with 2 µg/ml staurosporine (Biorbyt, Cambridge, United Kingdom) for 24 h. The treated and control cells were then harvested using 2x Laemmli buffer and analyzed by sodium dodecyl sulfate–polyacrylamide gel electrophoresis (SDS-PAGE) and western blotting.

### Antibodies and Chemicals

Primary antibodies against PARP1 and Bcl-XL were purchased from Santa Cruz (Dallas, USA), against actin from Sigma Aldrich (St. Louis, USA), and against Bax from BD Transduction Laboratories (BD Biosciences, Heidelberg, Germany). The antibody against *S. negevensis* GroEL (anti-Sn-GroEL) was prepared as previously described (Mehlitz et al., 2014). Inhibitors used: Z-VAD-FMK (50 µM) and Ac-YVAD-CMK (150 µM) (Invivogen, San Diego, USA), para-nitro-Blebbistatin (50 µM) (Cayman Chemical, Ann Arbor, USA), and Halt protease inhibitor cocktail (Thermo Fisher Scientific, Dreieich, Germany).

### Microscopy and Immunofluorescence

Immunofluorescence staining was performed as previously described (Mehlitz et al., 2010). Briefly, cells were seeded on glass cover slips of 15 mm in diameter (Paul Marienfeld, Lauda-Königshofen, Germany) and infected or re-infected for a respective time. After incubation, cells were fixed with 4% paraformaldehyde in PBS for 60 min. The fixed cells were stained using DAPI and an anti-*Sn*GroEL primary antibody. Pictures were recorded using LEICA DMR microscope.

### Western Blot

Lysates for western blot analysis were prepared by directly lysing cells in Laemmli buffer. Western blot analysis was performed as previously described (Mehlitz et al., 2010).

## Results

### *Simkania negevensis* Release of Infectious Particles From HeLa Cells Reaches Its Maximum 5 Days Post Infection and Is Coupled to the Loss of Infected Cells

**Figure 1:**
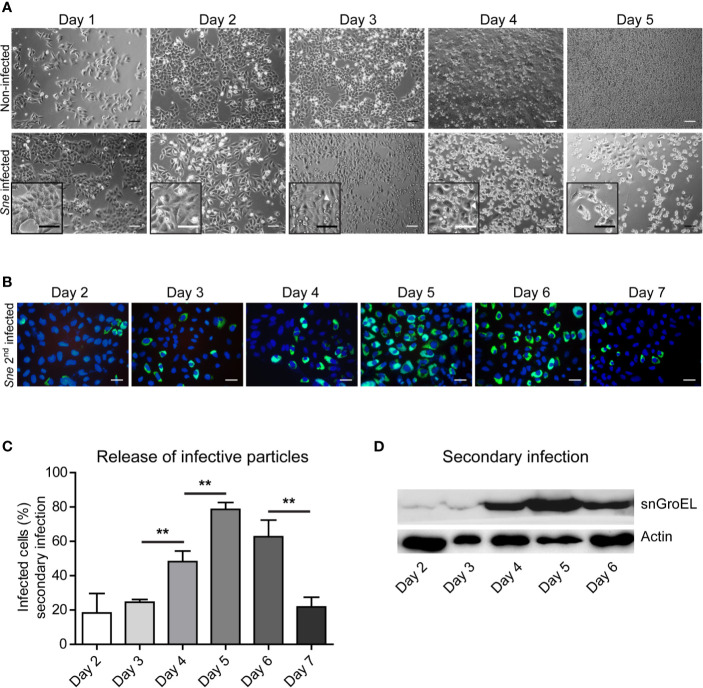
*Simkania*-infected HeLa loss correlates with the increase in the infectivity of the cell culture supernatant. **(A)** HeLa cells were infected with *S. negevensis* (MOI 1) in 12 well plates for 1 to 5 days. Non-infected cells were used as control. Pictures were recorded under phase contrast light microscope. Scale bar represents 100 μm. **(B)** HeLa cells were infected with *S. negevensis* (MOI 1) in 12 well plates for 2–7 days. The 24 h old supernatant was transferred to new cells for secondary infection, changed for fresh medium one day later and the infection was allowed to proceed for 3 days in total, when the cells were fixed and stained using DAPI and an anti-*S. negevensis* (Sn)GroEL primary antibody, followed by staining with fluorophore-coupled secondary antibody. Pictures were recorded using fluorescence microscopy. Scale bar represents 100 μm. **(C)** Cells from 6 random fields (2 fields per well of 3 repetitions in total) were counted under a 40× objective (per well at least 100 cells were counted). The graph shows the mean number of infected cells ± SD. The significance was assessed by Student’s t-test; **p ≤ 0.01. **(D)** The cell lysates of the secondary infection were analyzed by SDS-PAGE and western blot using primary antibodies against SnGroEL and as control against actin.

We first focused on epithelial cells as a model for *S. negevensis* infection. We infected cervical carcinoma HeLa cells with *S. negevensis* at MOI 1 and followed their fate during one week of infection. At the same time, we collected the medium from the infected cells every 24 h and used it to infect the second round of HeLa cells. The medium used for this secondary infection therefore always contained infectious particles released over the course of one day (**Figure 1**). Contrary to the observation made for the infection of Vero cells (Kahane et al., 2002), we could see that there was an increased cell loss in the culture of infected HeLa cells in comparison to the non-infected cells, which started on day 4 post infection (**Figure 1A**). Whereas the infectivity of the medium was relatively low up to 3 days post infection, on day 4 and especially day 5 post infection we observed a massive increase in the number of infectious particles, which resulted in the increase in secondary infection as shown by microscopy (**Figures 1B, C**) and western blot (**Figure 1D**). The medium from the infected cells showed a drop in the infectivity on day 6 and day 7 post infection, which was probably due to the sharp cell number reduction in the infected culture (**Figures 1B–D**). It appeared, therefore, that the infection of HeLa cells with *S. negevensis* resulted in cell detachment and/or lysis, which began on the day 4 post infection and correlated with the increase in the number of infectious particles in the cell culture medium.

### Release of *Simkania negevensis* Infectious Particles Is Reduced in the Presence of Caspase Inhibitors

We presumed that there was a connection between cell loss, cell death, and release of infectious particles of *S. negevensis* into the cell culture medium. To explore the role of caspases in this process, we treated the cells with increasing concentrations of pan-caspase inhibitor Z-VAD-FMK during primary infection. We then assessed the amount of released infectious particles by infecting the second round of HeLa cells, focusing on day 4 and day 5 post infection, when the release reached its maximum according to previous observations (**Figure 1**). We could observe the correlation between the increase in Z-VAD-FMK concentration and reduction in the number of infectious particles in the supernatant. Further increase of Z-VAD-FMK concentration beyond 50 µM seemed to have no effect (**Supplementary Figure**).

**Figure 2:**
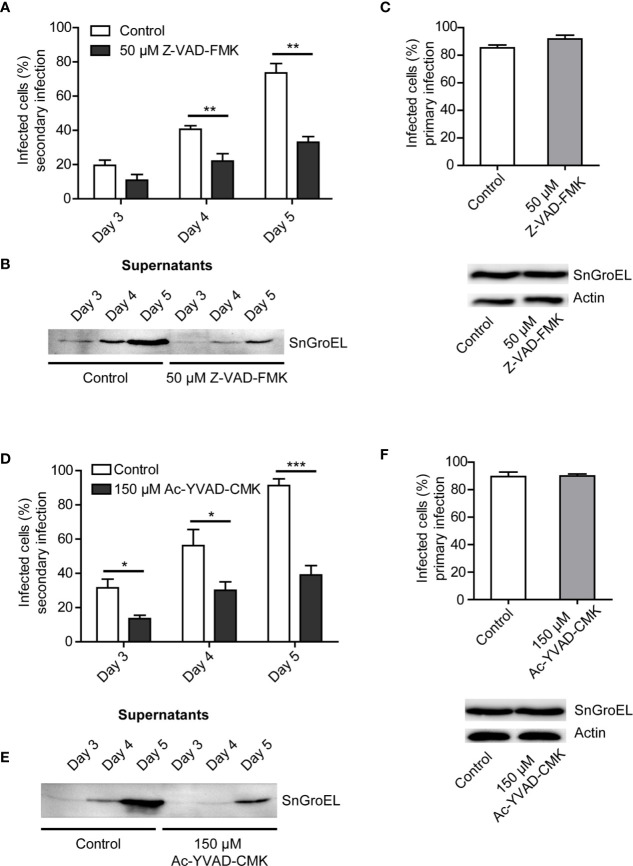
The rate of *S. negevensis* secondary infection decreases in the presence of Z-VAD-FMK and Ac-YVAD-CMK. **(A)** HeLa cells were infected in 12 well plates with *S. negevensis* (MOI 1) for 3, 4, and 5 days. DMSO (Control) and Z-VAD-FMK (50 μM) were added 6 h post infection. The 24 h supernatant was transferred to new cells, as described for **Figure 1**. Three days post secondary infection, the cells were fixed and stained using DAPI and an anti-SnGroEL primary antibody, followed by fluorophore-coupled secondary antibody staining and analyzed by fluorescence microscopy. Cells from 6 random fields (2 fields per well of 3 repetitions) were counted under a 40× objective (per well at least 100 cells were counted) and the percentage of infected cells was determined. Two independent experiments were performed (6 replicates in total). The graph shows the mean number of infected cells ± SD. The significance was assessed by Student’s t-test; **p ≤ 0.01. **(B)** HeLa cells were infected and treated as described in **(A)**. At the respective times, the supernatants were centrifuged, lysed, and analyzed by immunoblot. Primary antibody against SnGroEL (55 kDa) was used. **(C)** Investigation of the primary infection in the presence of Z-VAD-FMK. HeLa cells were infected with *S. negevensis* (MOI 1) in 12 well plates for 3 days. 6 h post infection DMSO (Control) and Z-VAD-FMK (50 μM) were added. The fixed cells were stained using DAPI and an anti-SnGroEL primary antibody. Pictures were recorded using fluorescence microscopy and cells from 6 random fields (2 fields per well of 3 repetitions in total) were counted under a 40× objective (per well at least 100 cells were included). The graph shows the mean number of infected cells ± SD. The cell lysates were analyzed by SDS-PAGE and western blot using primary antibodies against SnGroEL and as control against actin. **(D)** Statistical evaluation of the secondary infection rate in the presence of Ac-YVAD-CMK (150 μM). This was performed as described in **(A)**. *p ≤ 0.05; ***p ≤ 0.001. **(E)** Immunoblot analysis of the supernatants of infected cells in the presence of Ac-YVAD-CMK (150 μM) as previously described in **(B)**. **(F)** Investigation of the primary infection in the presence of 150 µM Ac-YVAD-CMK using immunofluorescence and immunoblot analysis. This was performed as described in **(C)**.

The repetition of Z-VAD-FMK treatment and analysis of secondary infection with supernatants from day 3 to day 5 post infection confirmed the negative effect of pan-caspase inhibitor on *Simkania* release (**Figure 2A**). We could also show that the supernatants of infected cell culture treated with Z-VAD-FMK contained a lower number of *S. negevensis* particles (**Figure 2B**). To exclude the effect of Z-VAD-FMK on primary infection, we incubated the infected cells for 3 days in the presence of the inhibitor and assessed the number of infected cells by microscopy of inclusions and western blot (**Figure 2C**). We conclude that Z-VAD-FMK does not inhibit infectivity or development of *S. negevensis* during primary infection, but reduces the release of infectious particles, pointing to a role of caspases in this process.

Z-VAD-FMK is a pan-caspase inhibitor. We wanted to test if the observed effect on *S. negevensis* release could be brought in connection with a specific caspase. To this purpose, we applied Ac-YVAD-CMK, an irreversible caspase 1 inhibitor. The addition of 150 µM Ac-YVAD-CMK to the cell culture medium strongly reduced the infectivity of the medium (**Figure 2D**), as well as the amount of *S. negevensis* particles in the cell supernatant as assessed by western blot (**Figure 2E**), without affecting primary infection (**Figure 2F**). These results indicated that caspase 1 plays a role in the release of *S. negevensis* from infected cells.

### Release of *Simkania negevensis* Infectious Particles From THP-1 Cells Follows a Similar Pattern as Observed for HeLa Cells

**Figure 3:**
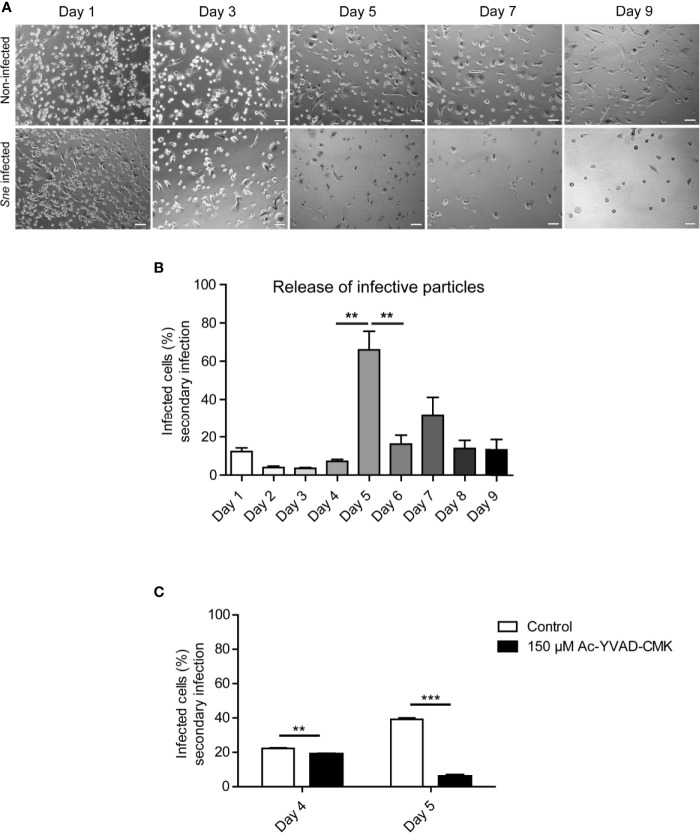
*S. negevensis* release from THP-1 cells reaches its maximum on day 5 post infection and is inhibited by Ac-YVAD-CMK. **(A)** THP-1 cells were differentiated in the presence of 20 ng/ml PMA and infected with *S. negevensis* (MOI 1) for 1–9 days. Non-infected differentiated THP-1 cells were used as control. Pictures were recorded under phase contrast light microscope. Scale bar represents 100 μm. **(B)** THP-1 cells as in A were infected with *S. negevensis* (MOI 1) in 12 well plates for 1–9 days. The 24 h old supernatant was transferred to HeLa cells for secondary infection, changed for fresh medium one day later and the infection was allowed to proceed for 3 days in total, when the cells were fixed and stained using DAPI and an anti-*S. negevensis* (Sn)GroEL antibody. Pictures were recorded using fluorescence microscopy. Cells from six random fields (two fields per well of three repetitions in total) were counted under a 40× objective (at least 100 cells were counted per well). The graph shows the mean number of infected cells ± SD. The significance was assessed by Student’s t-test; **p ≤ 0.01. **(C)** THP-1 cells were differentiated and infected as described for **(A, B)**. 3 days post infection 150 µM Ac-YVAD-CMK was added to the medium and the supernatant collected on days 4 and 5 post infection was used to infect a fresh round of HeLa cells. The graph represents the statistical evaluation of the secondary infection rate performed as described in **(B)**. **p ≤ 0.01; ***p ≤ 0.001.

THP-1 cells can be differentiated into macrophage-like cells in the presence of PMA and are a classical model for inflammasome and caspase 1 activation (Martinon et al., 2002). *S. negevensis* is capable of infecting these cells and actively replicating within them, unlike *C. trachomatis* (Herweg and Rudel, 2016). We therefore wanted to investigate if there was a difference in the dynamic of bacterial release from phagocytic in comparison to epithelial cells. We infected PMA-differentiated THP-1 cells with *S. negevensis* and followed both the cell numbers, as well as the infectivity of the supernatant over the course of 9 days. The number of infected differentiated THP-1 cells ranged from 60–70% (not shown). We observed that the total amount of THP-1 cells in the infected culture gradually decreased with time (**Figure 3A**). The infectivity of the cell culture supernatant was low up to day 5, when a strong increase was observed. The number of released infectious particles reduced on the subsequent days but remained somewhat higher than before day 5 (**Figure 3B**). Finally, we tested the effect of Ac-YVAD-CMK on the release of *S. negevensis* from differentiated THP-1 cells between days 4 and 5 post infection. To exclude the effect of Ac-YVAD-CMK on *S. negevensis* development, we added the inhibitor only after 3 days, when bacterial development had largely been completed. We observed that in the presence of Ac-YVAD-CMK the number of *S. negevensis* released in the supernatant on day 5 post infection was reduced even stronger than for HeLa cells (**Figure 3C**).

### *Simkania negevensis* Inhibits TNFα-, but Not Staurosporine-Induced Cell Death up to Day 3 Post Infection

**Figure 4:**
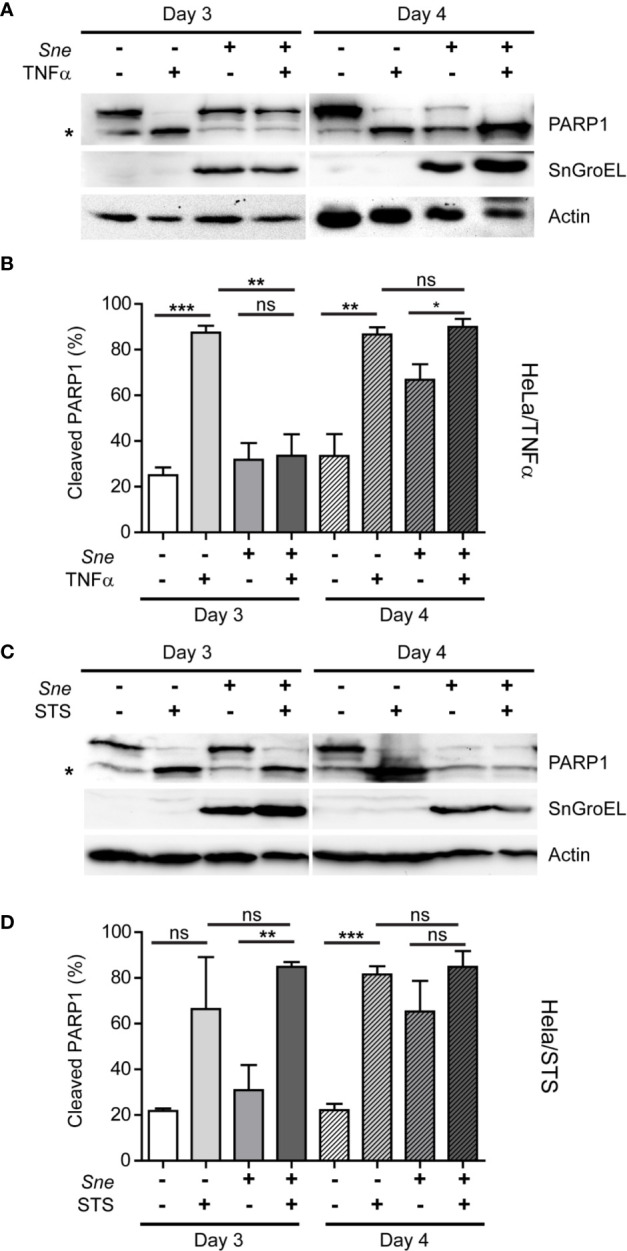
*S. negevensis* infection in HeLa inhibits TNF-α-, but not STS-induced apoptosis until day 3 post infection and induces cell death at later time points. **(A**, **D)** Cells were infected with *S. negevensis* (MOI 1) in 12 well plates for 3 and 4 days. Non-infected cells were used as control. Cells were treated with TNF- α (25 ng/ml) and CHX (5 μg/ml) for 12 h **(A, B)** and with STS (2 μg/ml) for 24 h **(C**, **D)**, lysed and analyzed by SDS-PAGE and western blot. For this, the antibodies against full-length (113 kDa) and cleaved (89 kDa) PARP1 (marked by asterisk), SnGroEL and actin as loading control were used. **(B, D)** Cleaved PARP1 was quantified relative to full-length PARP1 from four **(B)** and three **(D)** independent experiments using Fiji-Image J (Schindelin et al., 2012). The graphs show mean values ± SD. The significance was assessed by Student’s t-test; ns, not significant; *p ≤ 0.05; **p ≤ 0.01; ***p ≤ 0.001.

Previously, it has been shown that *S. negevensis* is capable of blocking programmed cell death (Karunakaran et al., 2011). Considering the connection between caspase activation and *Simkania*-induced cell lysis and release we observed, we wanted to explore in more detail the effect of infection on cell death induction. For this, we infected HeLa cells for 3 and 4 days, a time frame in which we registered an increased cell loss and infectivity of the cell culture supernatant, and induced cell death using TNFα in combination with CHX and staurosporine (STS). CHX was added because for the HeLa cell line we used, application of TNFα alone did not lead to cell death induction. TNFα induces the extrinsic pathway of apoptosis, which involves death receptors and caspase 8, whereas STS is a protein kinase inhibitor that induces apoptosis through an intrinsic pathway, depending on mitochondria and caspase 9 activation. Cell death induction was assessed by monitoring the amount of cleaved Poly(ADP-Ribose)-Polymerase 1 (PARP1), a DNA repair enzyme and a caspase substrate. We observed that *S. negevensis* effectively blocked TNFα-induced PARP1 cleavage on day 3, but not on day 4 post infection (**Figures 4A, B**). Surprisingly, when we applied STS as an inducer of cell death, we saw that *S. negevensis* could not inhibit PARP1 cleavage neither on day 3, nor on day 4 post infection (**Figures 4C, D**). At the same time, in non-treated infected samples we observed an increase in PARP1 cleavage on day 4 post infection, indicating cell death induction by *S. negevensis* at this point (**Figure 4**).

### The Release of *Simkania negevensis* Infectious Particles Does Not Depend on Bax, Bak, or Bcl-XL

**Figure 5:**
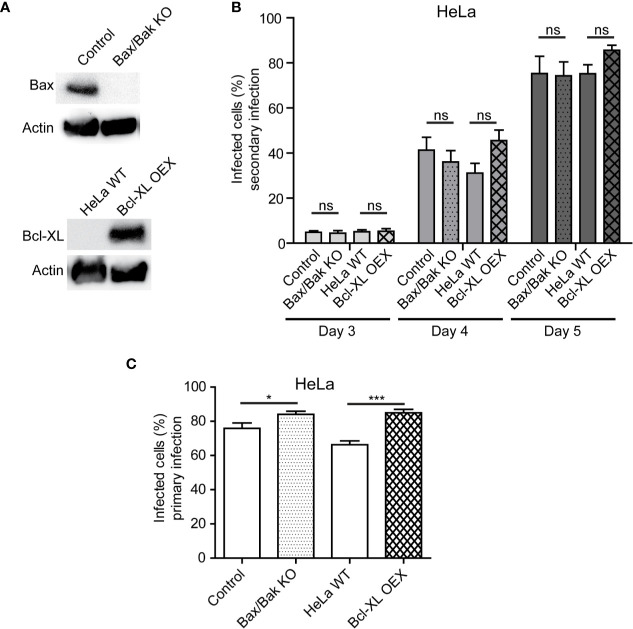
Bax, Bak, and Bcl-XL are not involved in release of *S. negevensis* particles from infected cells. **(A**, **B)** Bax/Bak double knockout cell line, Bcl-XL overexpressing stable cell line and respective control cell lines were infected with *S. negevensis* (MOI 1) in 12 well plates for 3–5 days. The knockout and overexpression were controlled by SDS-PAGE and western blot using antibodies against Bax, Bcl-XL and actin as control **(A)**. 24 h old supernatants were collected on day 3, 4, and 5 post infection and used to infect fresh HeLa cells, which were fixed 3 days later and stained with DAPI and snGroEL antibody, followed by decoration with fluorophore-coupled secondary antibody. Samples were analyzed by fluorescence microscopy and cells from six random fields (two fields per well of three repetitions in total) were counted under a 40× objective (per well at least 100 cells were included). The graph shows the mean number of infected cells ± SD. **(C)** Cells as in **(A)** were infected with *S. negevensis* (MOI 1) in 12 well plates for 3 days, fixed, stained with DAPI and SnGroEL antibody and respective fluorophore-coupled secondary antibody, and analyzed by fluorescence microscopy as described for **(B)** to assess the efficacy of the primary infection. The graph shows the mean number of infected cells ± SD. The significance was assessed by Student’s t-test; ns, not significant; *p ≤ 0.05; ***p ≤ 0.001.

As our experiments with STS implied that the intrinsic pathway of apoptosis was not controlled by *S. negevensis*, we wanted to explore how this was related to the release of *Simkania* infectious particles. The major role in the intrinsic pathway plays mitochondrial permeabilization, which is promoted by Bax and Bak pro-apoptotic, but inhibited by anti-apoptotic members of the Bcl-2 family, such as Bcl-XL (Peña-Blanco and García-Sáez, 2018). We therefore infected HeLa cells where Bax and Bak have been knocked out, as well as the cells overexpressing Bcl-XL (**Figure 5A**) (Brokatzky et al., 2019) with *S. negevensis* and compared the release of infectious particles to the respective control cell line. The lack of Bax/Bak or the overexpression of Bcl-XL had no effect on the infectivity of the cell culture supernatant on day 3, 4 and 5 post infection (**Figure 5B**). The primary infection with *S. negevensis* was also not affected (**Figure 5C**). This implied that Bax or Bak were not required by *S. negevensis* to effectively lyse the host cells and enable the release of the infectious particles into the environment.

### Myosin II and Proteases Play a Role in the Exit of *Simkania negevensis* From Host Cells

**Figure 6:**
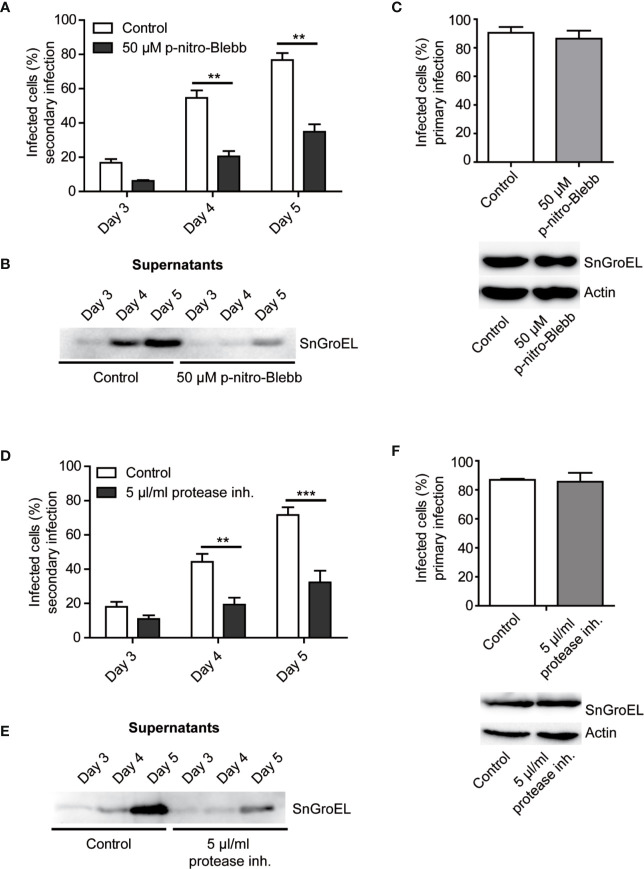
The rate of secondary infection by supernatant from *S. negevensis*-infected cells decreases in the presence of myosin II inhibitor and protease inhibitor cocktail. **(A**, **B)** HeLa cells were infected in 12 well plates with *S. negevensis* (MOI 1) for 3–5 days. DMSO (Control) and myosin II inhibitor para-nitro-Blebbistatin (p-nitro-Blebb) (50 μM) were added 6 h post infection. The 24 h old supernatants were transferred to new cells 3, 4, and 5 days later, and the secondary infection was allowed to proceed for 3 days, after which the cells were fixed and stained using DAPI and an anti-SnGroEL primary antibody, followed by staining with the respective fluorophore-coupled secondary antibody. Pictures were analyzed by fluorescence microscopy. Cells from six random fields (two fields per well of three repetitions in total) were counted under a 40× objective (a number of cells analyzed per well included at least 100 cells) and the percentage of infected cells was determined. The graph shows the mean number of infected cells ± SD. The significance was assessed by Student’s t-test; **p ≤ 0.01. At the respective time points, the supernatant was analyzed after centrifugation and lysis by immunoblot. A primary antibody against SnGroEL (55 kDa) was used **(B)**. **(C)** Investigation of the primary infection in the presence of p-nitro-Blebb. HeLa cells were infected with *S. negevensis* (MOI 1) in 12 well plates for 3 days. 6 h later DMSO (Control) and p-nitro-Blebb (50 μM) were added. Three days later, cells were fixed and stained using DAPI and an anti-SnGroEL primary antibody, plus the fluorophore-coupled secondary antibody. Pictures were made using fluorescence microscope and cells from 6 random fields (two fields per well of three repetitions in total) were counted under a 40× objective (per well included were at least 100 cells). The graph shows the mean number of infected cells ± SD. The cell lysates were analyzed by SDS-PAGE and western blot using primary antibodies against SnGroEL and as control against actin. **(D)** Statistical evaluation of the secondary infection rate in the presence of 5 µl/ml Halt protease inhibitor cocktail (protease inh.). The experiment was performed as described in **(A)**. **p ≤ 0.01. ***p ≤ 0.001. **(E)** Immunoblot analysis of the supernatant of infected cells in the presence of the Halt protease inhibitor cocktail as previously described for **(B)**. **(F)** Investigation of the primary infection in the presence of the Halt protease inhibitor cocktail using immunofluorescence and immunoblot analysis. This was performed as described in **(C)**.

In all previous experiments, the application of inhibitors or the absence of specific proteins reduced the number of released *S. negevensis* infectious particles but did not completely prevent it. This would indicate that the bacteria were capable of exiting host cell by another mechanism as well. Since the related bacteria *C. trachomatis* can get released from the host cell through a process called extrusion, which involves myosin II, we wanted to test if the same is the case for *S. negevensis*. To this purpose, we applied myosin II inhibitor para-nitro-blebbistatin and monitored the release of infectious particles from infected HeLa cells by using the supernatants collected on day 3 to day 5 post infection to infect the second round of HeLa cells. In the presence of para-nitro-blebbistatin the infection rate in the secondary infection dropped by more than half (**Figure 6A**), and the number of bacteria in the cell culture supernatant was strongly reduced (**Figure 6B**), even though the inhibitor did not significantly affect primary infection (**Figure 6C**). Similar results were obtained when we applied a broad-spectrum protease inhibitor (**Figures 6D–F**). The necessity for the proteases in *S. negevensis* exit is in concordance with the observed cell lysis, whereas the myosin II requirement indicates that *S. negevensis* might apply additional mechanisms for exiting infected cells, such as extrusion.

## Discussion

In this work, we endeavored to understand the mechanisms underlying the exit of *S. negevensis* from infected HeLa and THP-1 cells and the connection between cell death modulation and *Simkania* release. We observed several important features of *S. negevensis* infection, which included progressive loss of infected cells starting from day 3 post infection, number of infectious particles in the cell culture supernatant reaching its maximum on the day 5 post infection and importance of caspases for the described processes. *S. negevensis* could block programmed cell death until day 3 post infection, after which point the infection promoted cell death, reflected in the increased PARP1 cleavage. This process was not connected to mitochondrial permeabilization. We also showed that myosin II and proteases played a role in the release of *S. negevensis*.

Our results somewhat contradict the previous observations made by Kahane and colleagues (Kahane et al., 2002), considering that they did not observe significant cell lysis for the period of up to 12 days post infection and concluded that emergence of electron-dense forms of *S. negevensis* particles, increase in infectivity and host-cell lysis were not synchronized events. It is possible, however, to explain differences in the results by the fact that the dynamic of *S. negevensis* strongly depends on the infection conditions, dose, cell line used etc. In their work, the authors used Vero cells and performed infection in the presence of CHX, whereas we used HeLa and THP-1 cells and did not add CHX, to obtain conditions closer to the natural infection. It is possible that addition of CHX inhibits lysis of infected cells as has been observed for *C. trachomatis* (Yang et al., 2015). Also, we used relatively high MOIs with the rate of primary infection ranging from 60–90% depending on the experiment, which might have augmented the effects that would otherwise be less pronounced at lower MOIs. Importantly, even after more than a week of infection, we could still see infected cells among the remaining cells, but we were unable to determine if these were infected from the beginning or have been infected by *Simkania* released into the cell culture supernatant. Our results imply that in HeLa, as well as THP-1 cell infection, the release of infectious particles is a process synchronized at least in part with an increase in cell lysis (**Figures 1** and **3**) and correlates with the change in ability of *S. negevensis* to block cell death induction (**Figure 4**). Our results in this respect would correspond to the results of Vouga and colleagues, who showed that the number of *S. negevensis* particles from supernatants of infected Vero cells started increasing after 3 days of infection and was already at its maximum on day 6 post infection (Vouga et al., 2017). Unfortunately, the authors did not study in more detail the period between day 3 and day 6 post infection, which we could show is the time point of massive release of *S. negevensis* infectious particles (**Figure 1**).

In which way cell death manipulation, development and release from host cells are connected is a question important for many intracellular pathogens, including the related microorganism *C. trachomatis*, where this has been extensively studied (Sharma and Rudel, 2009). The role of caspases in the life cycle of *C. trachomatis* is somewhat disputed. The death of mouse embryonic fibroblasts infected by *C. trachomatis* could not be blocked by the inhibition of caspases using Z-VAD-FMK and neither was there evidence found for the activation of downstream caspase 3 (Ying et al., 2006), probably because, as later shown, *C. trachomatis* infection of mouse cells causes p53-mediated necrosis (Siegl et al., 2014). In human intestinal cells, on the other hand, it has been shown that caspase inhibition by Z-VAD-FMK and Ac-YVAD-CMK reduces cell mortality (Foschi et al., 2019). In Hep2 cells, the activation of caspase 3 at the later stages of *C. trachomatis* infection has also been documented (Matsuo et al., 2018). Our results for *S. negevensis* indicate that lower release of infectious particles, and therefore cell lysis, occurs when caspase inhibitors are present (**Figure 2**). It remains to be investigated, however, if the *S. negevensis*-induced cell death also shows hallmarks of necroptosis, as has been suggested for *C. trachomatis* infection of human intestinal cells due to the inhibition of cell mortality by necrostatin (Foschi et al., 2019) or necrosis, as has been described for the *C. trachomatis* infection of mouse fibroblasts (Siegl et al., 2014).

The role of mitochondria in cell death occurring after infection with *Chlamydia* and *Chlamydia*-related organisms shows not only some parallels but also differences. On one side, *C. trachomatis* preserves mitochondrial network and prevents its fragmentation by controlling the levels of p53 (Chowdhury et al., 2017). Further on, mitochondrial permeabilization and cytochrome *c* release in *Chlamydia*-infected cells is blocked in the initial phases of infection (Fan et al., 1998), but in the absence of Bax an Bak *C. trachomatis* is still able to cause nuclear fragmentation, indicative of cell death induction, at the end of infection cycle (Ying et al., 2006). It would imply that *C. trachomatis* controls mitochondrial cell death pathway in the beginning so as not to allow premature apoptosis induction but does not depend on it for the induction of cell lysis. *S. negevensis*, on the other hand, not only does not seem to be able to control intrinsic, mitochondria-mediated apoptosis induction by STS (**Figures 4C, D**) but also does not depend on mitochondrial permeabilization and Bax and Bak for cell lysis and release (**Figures 5A–C**). This could be the result of the specificities in the development of *S. negevensis*, which primarily induces ER stress and therefore has to inhibit it to prevent premature cell death (Mehlitz et al., 2014). Mitochondria might be, therefore, marginal in this process. For another environmental *Chlamydia*, *Parachlamydia acanthamoebae*, which are unable to control mitochondria-mediated apoptosis induction in infected HeLa cells, the growth is only possible in the absence of Bax and Bak (Brokatzky et al., 2020). Therefore, despite common denominators, differences nevertheless exist in the way different *Chlamydia*-like microorganisms manipulate host cell death.

Although *C. trachomatis* can block apoptosis and downstream caspase activation upon pro-apoptotic insults, infected cells are still susceptible to non-canonical necrosis that can be induced by STS or TNFα (Sixt et al., 2019). *S. negevensis* shows in this aspect differences because we observe that the cleavage of PARP1, a substrate of downstream caspases, is blocked by *Simkania* only upon TNFα, but not STS induction of cell death. This blockage also disappears after 3 days of infection, and cell death seems to be supported by *S. negevensis* from that point on, probably as a release mechanism (**Figure 3**). This data contradicts partially the report by Karunakaran and colleagues, which showed that *Simkania* infection significantly decreased the number of apoptotic cells in STS-treated samples (Karunakaran et al., 2011). In this work, however, only the number of morphologically changed cells upon Hoechst staining was counted, but PARP1 cleavage was not assessed. Our data however resemble the situation seen upon infection of cells with *Waddlia chondrophila*, a Chlamydiales member that is also unable to inhibit STS-induced cell death (Dille et al., 2015).

The involvement of proteases in the release of *S. negevensis* is implied by the negative effect of protease inhibitors on the number of infective particles in the cell culture supernatant and would support the role of host cell lysis in this process (**Figure 6**). Contrary to *Chlamydia*, where cysteine protease inhibitor E64 strongly inhibited host cell lysis (Hybiske and Stephens, 2007), we saw no such effect on *Simkania*, at least as assessed by the infectivity of the cell culture supernatant (not shown), which could mean that serine or other types of proteases play a more pronounced role. Finally, similar to *C. trachomatis* (Hybiske and Stephens, 2007), inhibition of myosin II by blebbistatin strongly reduced the release of infectious particles (**Figure 6**), which would point to a possibility of an extrusion playing a role in this process. We were, however, so far unable to visualize the extrusion of *Simkania* as has been demonstrated for *Chlamydia* (Hybiske and Stephens, 2007). Additional experiments, including visualization and counting of extrusions or usage of other specific inhibitors and inducers of cytoskeletal function to confirm extrusion and exclude off-target effects of blebbistatin are essential for clarifying this point.

In conclusion, the exit of *S. negevensis* from infected HeLa cells depends on cell lysis mediated by caspase activation, including more specifically caspase 1 and proteases. Our experiments indicate a possible role of extrusion, as well, or at least the involvement of myosin II. It remains to be seen if *S. negevensis* relies on pyroptotic signaling for cell lysis, or if necrosis plays a significant role. Likewise, it would be interesting to further explore the involvement of TNF receptor signaling in cell death induced by infection with *Simkania*. *C. trachomatis* are known to express *Chlamydia* protein associating with death domains (CADD) late in infection, a protein with homologues in other *Chlamydia* species, which interacts with TNF family receptors and induces cell death (Stenner-Liewen et al., 2002). It is possible that *Simkania* manipulate cell death for their own benefit in a similar way. In addition, it is apparent that the dynamic of *S. negevensis* infection also depends on the cell type which serves as a host. In the future, all these aspects need to be explored to gain a complete picture of the release of *S. negevensis* from infected cells.

## Data Availability Statement

The original contributions presented in the study are included in the article/**Supplementary Material**. Further inquiries can be directed to the corresponding author.

## Author Contributions

R-DK, E-MH, NM, EM, and VK-P performed experiments. R-DK and VK-P designed experiments, analyzed the data, and wrote the manuscript. VK-P provided financial support for the project. All authors contributed to the article and approved the submitted version.

## Funding

This work was funded by the GRK 2157 and GRK 2581 to VK-P. This publication was funded by the German Research Foundation (DFG) and the University of Wuerzburg in the funding program Open Access Publishing.

## Conflict of Interest

The authors declare that the research was conducted in the absence of any commercial or financial relationships that could be construed as a potential conflict of interest.
