# Supplementary material for: Modulation of Host Cell Death and Lysis Are Required for the Release of Simkania negevensis
Source: Front Cell Infect Microbiol. 2020 Oct 29;10:594932. doi: 10.3389/fcimb.2020.594932 (PMC7658264; doi:10.3389/fcimb.2020.594932)
Supplement: Supplementary file 1 [file DataSheet_1.docx]

Supplementary Material


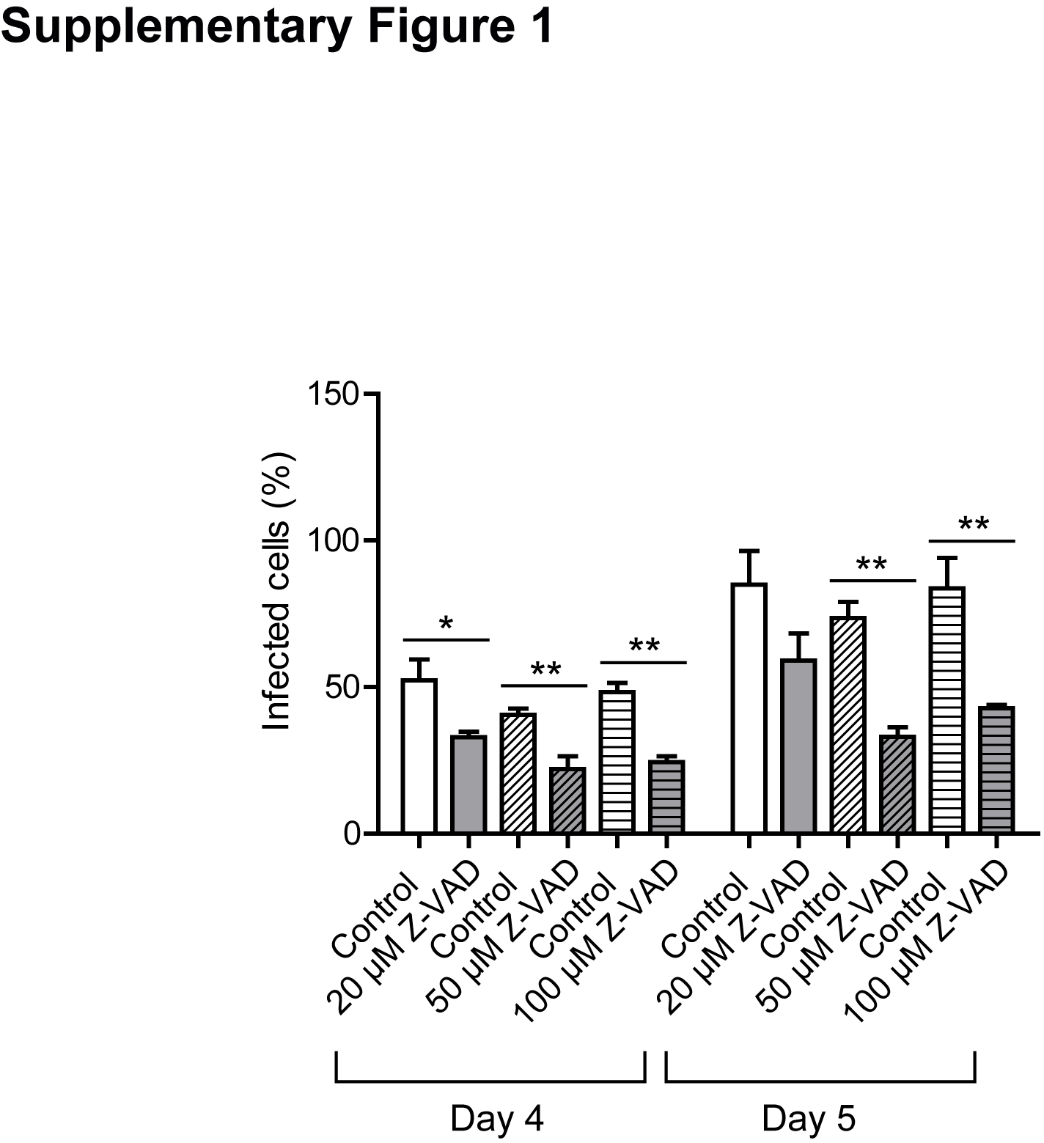


**Supplementary Figure 1.** Z-VAD-FMK decreases the number of infective particles in supernatants of *S. negevensis*-infected cells in a dose-dependent way. HeLa cells were infected in 12 well plates with *S. negevensis* (MOI 1) for 4 and 5 days. DMSO (Control) and Z-VAD-FMK (20 μM, 50 μM and 100 μM) were added 6 hours post infection. The supernatant was transferred to new cells, which were fixed after 3 days and stained using DAPI and an anti-*Sn*GroEL primary antibody, followed by decoration with the fluorophore-coupled secondary antibody. Pictures were recorded using fluorescence microscopy. Cells from 6 random fields (2 fields per well of 3 repetitions in total) were counted under a 40x objective (per well at least 100 cells were counted) and the percentage of infected cells was determined and shown on the graph as a mean value ± SD. The significance was calculated using Student’s t-test. *p≤0.05; **p≤0.01.
